# Physical activity in older cancer patients: evidence and clinical implications

**DOI:** 10.3389/fonc.2025.1613268

**Published:** 2025-08-18

**Authors:** Shugo Yajima, Shin Kobayashi, Tadayoshi Hashimoto, Hitoshi Masuda

**Affiliations:** ^1^ Department of Urology, National Cancer Center Hospital East, Kashiwa, Japan; ^2^ Perioperative Treatment Development Promotion Office, National Cancer Center Hospital East, Kashiwa, Japan

**Keywords:** cancer, elderly, exercise, survivorship, cancer prevention, rehabilitation, quality of life

## Abstract

This mini-review examines the role of physical activity in older cancer patients based on current evidence. As cancer incidence increases with age, older patients face unique challenges including comorbidities and functional decline. This review synthesizes findings regarding physical activity’s impact on cancer prevention, post-operative recovery, and long-term outcomes in older adults. Evidence consistently demonstrates that moderate physical activity reduces risk for several cancers, improves post-surgical recovery, and enhances quality of life while potentially improving survival in cancer survivors. We also address the independent risks of sedentary behavior and briefly discuss alternative forms of physical activity such as yoga and tai chi that may be suitable for older adults. While optimal exercise regimens for specific populations remain under investigation, this review provides evidence-based guidance for incorporating physical activity across the cancer care continuum, emphasizing approaches that account for age-related considerations and functional status of older adults.

## 1 Introduction

Cancer incidence increases with age, and older cancer patients face unique challenges including multiple comorbidities and age-related functional declines such as sarcopenia and frailty (1, 2). These factors complicate treatment decisions, recovery, and long-term prognosis.

A paradigm shift has occurred in oncology practice, with physical activity now recognized as essential throughout the cancer care continuum (3, 4). This approach represents a profound change from earlier recommendations of rest to the current understanding that appropriate activity offers multiple benefits for cancer patients and survivors (5). The American College of Sports Medicine (ACSM), American Cancer Society (ACS), and World Health Organization (WHO) now advocate for physical activity as a standard component of cancer prevention and treatment (**Table 1**) (6–8). Specifically, the ACS recommends that adults engage in 150–300 minutes of moderate-intensity or 75–150 minutes of vigorous-intensity aerobic physical activity per week, with similar recommendations adapted for cancer survivors based on their functional capacity (7).

**Table 1:** Summary of key physical activity guidelines for older adults and cancer survivors.

Guideline Source	Aerobic Activity Recommendation	Resistance Training Recommendation
American College of Sports Medicine (ACSM) (6)	Provides specific FITT prescriptions based on the health outcome being targeted (e.g., fatigue, anxiety, physical function). A general goal is 3x/week for 30 min sessions.	Provides specific FITT prescriptions based on health outcomes. A general goal is 2x/week, with 2 sets of 8–15 reps.
American Cancer Society (ACS) (7)	150–300 min/week of moderate-intensity OR 75–150 min/week of vigorous-intensity.	N/A
World Health Organization (WHO) (8)	150–300 minutes/week of moderate-intensity OR 75–150 minutes/week of vigorous-intensity aerobic activity	Two or more days/week of moderate or greater intensity muscle-strengthening activities involving all major muscle groups

FITT, Frequency, Intensity, Time, Type; N/A, not available.

**Figure 1:**
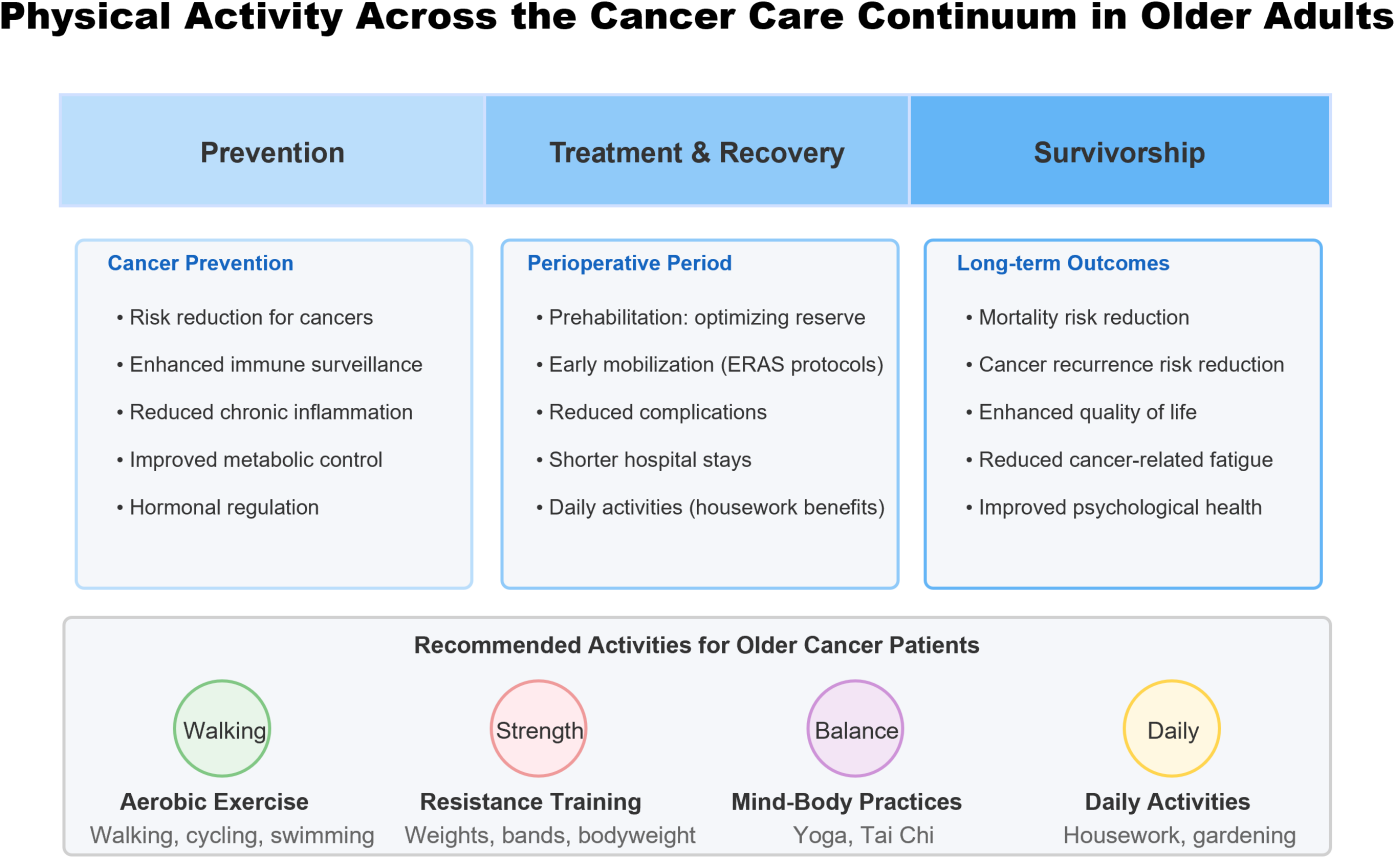
Physical activity across the cancer care continuum for older adults with cancer.

This mini-review focuses on older cancer patients, examining evidence for physical activity’s impact on cancer prevention, post-operative recovery, and long-term outcomes. **Figure 1** provides a comprehensive overview of physical activity’s role across the cancer care continuum in older adults.

While this review synthesizes international evidence, it is important to acknowledge that cultural and socioeconomic factors significantly influence physical activity preferences, adherence, and access. In Japan, for instance, group-based exercises and activities integrated into daily routines (such as radio calisthenics) are culturally embedded. In contrast, Western populations may emphasize structured, individualized gym-based programs. Beyond cultural preferences, health equity issues are paramount; socioeconomic status, educational attainment, and geographic location (e.g., rural versus urban residence) can create significant disparities in access to safe environments for activity and specialized programs. Understanding these diverse socio-cultural and economic contexts is critical for designing and implementing effective, equitable, and globally applicable interventions. The evidence for this mini-review was compiled through a non-systematic search of the PubMed, Google Scholar, and Scopus databases for articles published up to January 2025. Search terms included combinations of “physical activity,” “exercise,” “cancer,” “elderly,” “older adults,” “geriatric,” “prevention,” “survivorship,” “rehabilitation,” “sedentary behavior,” and “quality of life.” Preference was given to systematic reviews, meta-analyses, major prospective cohort studies, and recent clinical trials. The selection of sources aimed to provide a comprehensive overview of the current evidence base relevant to older cancer patients.

## 2 Physical activity and cancer prevention in older adults

### 2.1 Epidemiological evidence

Consistent evidence from large-scale prospective studies, including a major pooled analysis (9), indicates that individuals with higher levels of leisure-time physical activity experience a significantly lower risk—generally ranging from 10% to over 20%—for numerous cancers such as bladder, breast, colon, endometrial, esophageal adenocarcinoma, and kidney cancer, compared to those with the lowest activity levels. The Japan Public Health Center-based Prospective Study (JPHC Study) found that higher total daily physical activity was associated with lower overall cancer incidence (13% reduction in men and 16% reduction in women when comparing highest versus lowest activity groups) (10).

Further strengthening this evidence with objective measurements, a recent prospective analysis of the UK Biobank cohort using wrist-worn accelerometers found that greater total daily physical activity was significantly associated with a lower risk of developing a composite of 13 physical-activity-related cancers (HR per 1 SD increase: 0.85, 95% CI 0.81–0.89) (11).

### 2.2 Biological mechanisms

The protective effects of physical activity against cancer development and progression are underpinned by a complex interplay of systemic and local biological modifications. While human studies provide correlative evidence, preclinical research, particularly utilizing murine models, has been instrumental in elucidating specific molecular and cellular pathways involved.

Enhanced Immune Surveillance and Modulation of the Tumor Microenvironment (TME): Physical activity robustly impacts anti-tumor immunity. Exercise has been shown to mobilize and enhance the trafficking and cytotoxic activity of immune cells crucial for tumor elimination, such as Natural Killer (NK) cells and CD8+ T cells. For instance, studies in mouse models demonstrate that voluntary wheel running can increase NK cell infiltration into tumors and impede tumor growth, partly mediated by exercise-induced epinephrine release which primes NK cells via IL-6 signaling from other immune cells (12). Furthermore, exercise demonstrably remodels the tumor immune microenvironment, critically enhancing the role of cytotoxic T lymphocytes. The work by Rundqvist et al. established in murine cancer models that voluntary exercise significantly increased the frequency of intratumoral CD8+ T cells, and crucially, that the exercise-induced suppression of tumor growth was dependent upon this CD8+ T cell population, as its depletion abrogated the beneficial effect (13). It is important to note that the aging process itself can alter the tumor microenvironment, for example, by increasing the burden of senescent cells which contribute to a pro-inflammatory state. Exercise may counteract these age-specific changes, although this is a developing area of research. While these preclinical findings are compelling, clinical translation is still evolving.

Attenuation of Chronic Inflammation: Chronic inflammation fosters a pro-tumorigenic environment, a condition that physical activity may effectively counteract through its anti-inflammatory properties. Preclinical validation is provided by studies such as one employing a murine model of breast cancer, where regular endurance exercise training significantly mitigated the elevated systemic levels of key inflammatory mediators MCP-1 and IL-6 observed during tumor development. Importantly, this dampening of systemic inflammation coincided with significantly reduced tumor growth, indicating that the attenuation of these inflammatory pathways likely contributes to the anti-cancer benefits of exercise (14).

Metabolic Reprogramming and Hormonal Regulation: Exercise exerts profound effects on host metabolism, potentially creating a systemic and local environment less permissive for cancer growth. A key mechanism involves enhanced systemic metabolic control; regular physical activity improves insulin sensitivity and can decrease circulating levels of insulin and Insulin-like Growth Factor 1 (IGF-1). This improved metabolic profile subsequently dampens the activation of critical pro-proliferative signaling cascades, such as the PI3K/Akt/mTOR pathway (15). Concurrently, exercise modulates the TME itself. By promoting normalization of tumor vasculature, physical activity can improve perfusion, thereby potentially enhancing crucial energy substrate availability while reducing the accumulation of metabolic byproducts like lactate within the TME (16). Furthermore, exercise influences hormonal balance, a factor particularly relevant for hormone-sensitive malignancies. While long-term training is often associated with reduced basal concentrations of circulating sex hormones (15), the interaction appears complex. For instance, in a chemically-induced rat model of hormone-receptor-positive breast cancer, exercise training inhibited metastasis despite causing an increase in circulating estrogen levels, suggesting that exercise can also exert protective, hormone-independent effects (17). Clinical trials in cancer survivors have largely corroborated these metabolic benefits, demonstrating that structured exercise can improve insulin sensitivity and reduce circulating IGF-1 levels (18). However, data specifically from frail or very old patient cohorts remain limited, representing a key knowledge gap.

## 3 Post-operative recovery and daily activities

### 3.1 Pre- and post-operative exercise

Prehabilitation, defined as interventions implemented between cancer diagnosis and the commencement of acute treatment such as surgery, aims to optimize physiological reserve and potentially enhance surgical outcomes (19). While evidence supporting its efficacy is growing, particularly for multimodal approaches, its impact across all postoperative endpoints requires further elucidation. A recent systematic review and meta-analysis focusing on abdominal cancer surgery indicated that multimodal prehabilitation, typically combining exercise, nutritional optimization, and psychological support, significantly improved preoperative functional capacity, evidenced by increased 6-minute walk distance, and reduced hospital length of stay (19). However, this analysis did not find a statistically significant reduction in overall postoperative complications associated with prehabilitation interventions compared to standard care.

Postoperatively, early mobilization is emphasized as a critical component of contemporary recovery strategies, such as Enhanced Recovery After Surgery (ERAS) protocols (20). The prompt initiation of physical activity, particularly ambulation, is advocated based on established physiological benefits: stimulating intestinal peristalsis to reduce the risk of ileus, promoting effective lung ventilation thereby preventing respiratory complications, and enhancing systemic blood flow, which serves to mitigate the risk of venous thromboembolism (20).

### 3.2 Daily activities and sedentary behavior

Prolonged sedentary time—characterized by minimal energy expenditure (≤1.5 Metabolic Equivalents [METs]) during waking hours while sitting, reclining, or lying—constitutes a distinct health risk factor, independent of overall physical activity levels (21). Robust evidence synthesized through meta-analyses and systematic reviews demonstrates that extensive sedentary behavior is associated with an increased incidence of several cancers, including endometrial, colorectal, and breast cancer, as well as elevated overall cancer mortality (21, 22).

Conversely, integrating even light-intensity physical activity (1.6-2.9 METs) (23) into daily routines may hold potential benefits. Emerging research exploring routine activities, such as household chores, suggests a possible association between preoperative housework participation and improved survival outcomes among male patients following urologic cancer surgery (24). To mitigate risks, guidelines recommend strategies for reducing and interrupting sedentary time. Emerging evidence suggests that regularly interrupting prolonged sitting bouts with brief periods of standing or light ambulation is an evidence-based strategy that may improve metabolic health and is a particularly achievable goal for older adults with functional limitations (25). Key recommendations include consciously incorporating incidental activity via household tasks, regularly interrupting periods of extended sitting with brief standing or ambulation, utilizing stairs instead of elevators when practical, and prioritizing active rather than passive leisure-time pursuits (26).

## 4 Physical activity in cancer survivorship

### 4.1 Impact on survival and recurrence

A substantial body of evidence indicates that engaging in physical activity following a cancer diagnosis significantly influences patient prognosis (27, 28). Comprehensive systematic reviews and meta-analyses consistently demonstrate that cancer survivors achieving higher levels of post-diagnosis physical activity experience markedly lower mortality rates compared to their less active counterparts. Specifically, pooled analyses reveal statistically significant risk reductions of approximately 37-39% for all-cause mortality and 37% for cancer-specific mortality across various cancer types when comparing the most active survivors to the least active (28). Reduced risk of cancer recurrence has also been strongly associated with higher physical activity levels (27).

### 4.2 Quality of life and symptom management

Beyond impacting survival, physical activity confers significant benefits for the health-related quality of life (HRQoL) among cancer survivors. Systematic reviews, including a comprehensive Cochrane analysis, indicate that structured exercise interventions can yield positive effects on overall HRQoL and various domains such as emotional well-being, sleep disturbance, social functioning, anxiety, and pain (29). Specific modalities like yoga have also demonstrated improvements in overall quality of life and psychosocial well-being (30). Furthermore, physical activity is recognized as an effective strategy for managing cancer-related fatigue (CRF)—a persistent exhaustion not adequately alleviated by rest. Network meta-analyses comparing non-pharmacological approaches confirm that various forms of exercise (aerobic, resistance, or combined), alongside interventions like relaxation and yoga, represent key evidence-based strategies for mitigating CRF during and after cancer treatment (31).

Regarding psychological health, exercise interventions can positively impact mood and alleviate distress. A meta-analysis focused on depressive symptoms established that exercise yields modest yet significant reductions among cancer survivors (32, 33). Potential benefits for reducing anxiety have also been reported in systematic reviews evaluating exercise and yoga interventions (29, 30). Particularly for older cancer survivors, improvements in these psychosocial dimensions can substantially enhance overall quality of life. Beyond mood, physical activity may bolster neurocognitive function (34). There is growing evidence that exercise can improve domains like executive function in cancer survivors, which is critical for maintaining independence.

## 5 Alternative physical activity approaches

For individuals, particularly older cancer survivors, who may find conventional forms of exercise challenging or less appealing, alternative mind-body practices such as yoga and Tai Chi represent viable options for maintaining physical activity (7, 36). Yoga, characterized by its integration of physical postures, breathing techniques, and meditation, has demonstrated notable benefits in cancer populations. Systematic reviews and meta-analyses indicate that yoga participation can significantly improve psychological outcomes, including reductions in anxiety, depression, and perceived stress, while also enhancing overall health-related quality of life and potentially alleviating cancer-related fatigue (30, 35). Tai Chi is another commonly utilized mind-body practice among survivors (31, 36), although evidence detailing its specific effects within diverse cancer populations requires further investigation. These approaches are often low-impact and adaptable, making them potentially suitable for a wide range of capabilities; however, more research is warranted to fully delineate their benefits, particularly among older cancer survivors.

## 6 Discussion, future directions, and conclusion

The evidence reviewed establishes physical activity as a valuable intervention across the cancer care continuum for older adults. From prevention to survivorship, appropriate activity offers substantial benefits. However, translating this evidence into practice requires addressing conceptual frameworks, patient heterogeneity, and systemic barriers.

### 6.1 A conceptual framework: integrating aging biology and physical activity

The profound benefits of physical activity in older cancer patients can be framed through the lens of geroscience, which posits that targeting the fundamental biological processes of aging can prevent or mitigate age-related diseases like cancer. Exercise directly counteracts several “hallmarks of aging” (37). For instance, it enhances mitochondrial function, reduces the burden of senescent cells, improves nutrient-sensing pathways (e.g., dampening mTOR signaling), and attenuates chronic inflammation—all of which are implicated in both aging and cancer progression. By intervening in these core pathways, physical activity may not only manage cancer-related outcomes but also improve an older individual’s overall physiological resilience and “intrinsic capacity” as defined by the WHO (8).

### 6.2 Barriers to implementation in geriatric oncology

Despite strong evidence, implementation challenges persist, particularly for older adults. Significant barriers at the patient level include the high prevalence of geriatric syndromes such as sarcopenia, frailty, cognitive decline, polypharmacy, and severe comorbidities, which can make conventional exercise difficult. Psychosocial barriers are also common, including fear of injury, pain, fatigue, low self-efficacy, and lack of social support. At the healthcare system level, structural barriers are a major impediment. These include a lack of provider education on exercise prescription, insufficient time during clinical encounters for counseling, and a lack of standardized, reimbursed referral pathways to qualified professionals such as physiotherapists or certified cancer exercise trainers.

### 6.3 Tailoring physical activity for heterogeneous older adults: the role of precision prescription

Older adults are not a monolith; their functional status ranges from robust to frail. A “one-size-fits-all” approach is inadequate. Precision exercise prescription is needed, tailored to tumor type, treatment phase, comorbidities, and frailty phenotype. Clinical assessment tools can guide this process. For example, the Short Physical Performance Battery (SPPB) (38) can objectively measure physical function and help stratify patients to determine a safe and effective starting intensity. Geriatric assessment tools like the Cancer and Aging Research Group (CARG) score (39) can identify vulnerabilities that may require modifications to an exercise plan. For frail individuals, the initial focus may be on low-intensity functional exercises to build a foundation and prevent falls, before progressing to moderate-intensity aerobic or resistance training.

### 6.4 Future directions and conclusion

Key future research priorities must address these challenges. These include:

- Studies specifically targeting the oldest-old (≥75 years) and frail populations to determine optimal, safe exercise regimens.
- Research on the durability of benefits and strategies to promote long-term adherence after structured interventions end.
- Investigation of digital health technologies for remote monitoring and support.
- Implementation science to integrate standardized assessment and referral pathways into routine cancer care.
- Economic analyses to justify the value proposition of exercise programs to policymakers and healthcare systems.

Healthcare professionals should incorporate physical activity assessment and counseling as standard components of geriatric oncology care. In conclusion, current evidence strongly supports physical activity as an integral component of comprehensive cancer care for older adults, offering a non-pharmacological intervention that enhances physiological outcomes and quality of life.

This schematic illustrates the beneficial effects of physical activity at each stage of the cancer care continuum. Physical activity provides multiple benefits during prevention (reducing cancer risk through immune enhancement and metabolic regulation), perioperative period (optimizing physiological reserve and facilitating recovery), and survivorship (improving both quantity and quality of life). Recommended activity modalities for older cancer patients include aerobic exercise, resistance training, mind-body practices, and daily activities, all of which should be tailored to individual functional capacity and medical conditions.
